# Local Intramedullary Delivery of Vancomycin Can Prevent the Development of Long Bone *Staphylococcus aureus* Infection

**DOI:** 10.1371/journal.pone.0160187

**Published:** 2016-07-29

**Authors:** Catherine Loc-Carrillo, Caroline Wang, Ahranee Canden, Michael Burr, Jayant Agarwal

**Affiliations:** 1 VA Salt Lake City Health Care System, Salt Lake City, Utah, United States of America; 2 Division of Plastic and Reconstructive Surgery, Department of Surgery, University of Utah School of Medicine, Salt Lake City, Utah, United States of America; 3 Department of Ophthalmology and Visual Sciences, Moran Eye Center, University of Utah School of Medicine, Salt Lake City, Utah, United States of America; Chang Gung Memorial Hospital, TAIWAN

## Abstract

Current treatments for methicillin-resistant *Staphylococcus aureus* (MRSA) infections require intravenously delivered vancomycin; however, systemically delivered vancomycin has its problems. To determine the feasibility and safety of locally delivering vancomycin hydrochloride (~25 mg/Kg) to the medullary canal of long bones, we conducted a pharmacokinetics study using a rat tibia model. We found that administering the vancomycin intraosseously resulted in very low concentrations of vancomycin in the blood plasma and the muscle surrounding the tibia, reducing the risk for systemic toxicity, which is often seen with traditional intravenous administration of vancomycin. Additionally, we were able to inhibit the development of osteomyelitis in the tibia if the treatment was administered locally at the same time as a bacterial inoculum (i.e., Log_10_ 7.82 CFU/mL or 6.62x10^7^ CFU/mL), when compared to an untreated group. These findings suggest that local intramedullary vancomycin delivery can achieve sufficiently high local concentrations to prevent development of osteomyelitis while minimizing systemic toxicity.

## Introduction

*Staphylococcus aureus* is the most common bacteria causing bone infection [1] and the primary pathogen associated with orthopedic surgical site infections [2]. When bone becomes infected with *S*. *aureus*, inflammatory factors and leucocytes can lead to tissue damage, a restriction in blood supply (i.e., ischemia), and destruction of trabecular bone and bone matrix [3]. Segments of necrotic bone (i.e., sequestra) separate from healthy bone and allow bacteria to reside within this avascular area. However, when treated promptly, acute osteomyelitis can be halted prior to progression to chronic osteomyelitis and bone necrosis [4].

Surgical site infection (SSI) is a complication that occurs in 2–5% of surgeries in the United States and result in a 300% increase in the cost of treatment [5, 6]. Surgical site infections involving orthopaedic surgery have an even greater negative impact resulting in increased morbidity, mortality, and economic burden. Risk factors associated with SSI include patients who are less fit, have a greater in-hospital exposure time, and undergo longer more complex surgeries [5]. To reduce infection rates surgeons use antibiotic prophylaxis as a preventative treatment [7, 8].

Due to the increase in methicillin resistant *Staphylococcus aureus* (MRSA) infections, the use of vancomycin in U.S. hospitals has been on the incline since its initial discovery over 60 years ago [9, 10]. Vancomycin is a broad-spectrum antibiotic, which is able to penetrate most body tissues and one of the few antibiotics that is effective against MRSA [11]. Vancomycin is typically administered intravenously; however, systemic delivery of antibiotics results in low local concentrations in or near the bone [2]. Recent research has shown that the use of vancomycin powder at the surgical site has been shown to reduce the incidence of infection [12]. However, delivering vancomycin powder onto a surgical wound can be difficult to distribute evenly and can make it challenging to determine its concentration at the desired location.

Currently prophylactic vancomycin is administered systemically and due to increasing minimum inhibitory concentrations (MIC) in target pathogens, dosing concentrations of vancomycin are becoming more aggressive. High plasma concentration of vancomycin and its prolonged use has been associated with an increased risk in nephrotoxicity [11, 13]. As an alternative, local administration of antibiotic at, higher doses can provide the necessary therapeutic concentrations of drug to kill the target bacteria causing infection, while containing the potential toxigenic effects of the drug, which occur when administered systemically.

Drug delivery systems are generally designed to release a drug locally over a prolonged period of time [14–16], but there is a growing consensus that the duration of antibiotic therapy should be reduced to reduce adverse drug reactions and the development of antibiotic-resistant infections [17, 18]. In addition, the use of biomaterials that are bioinert or non-degradable require a revision surgery to remove the implants, which can be both costly and painful. The purpose of this study is to demonstrate the safety and efficacy of intramedullary delivery of aqueous vancomycin to prevent long bone infection. We intend to show the efficacy of a single-dose of locally delivered vancomycin to the site of bacterial inoculation can produce sustained high local concentrations without high systemic concentration and prevent the development of infection.

## Materials and Methods

### Animals

Male Sprague-Dawley rats (Charles River Lab Inc. Wilmington, MA) > 9 weeks old and > 500 grams in weight were housed in a controlled environment with regulated temperature, 12-h light/dark cycle, and were allowed food and water *ad libitum*. The Salt Lake City VA’s Institutional Animal Care and Use Committee (IACUC) approved the animal use and experimental protocol, prior to commencing the study (ACORP 13–01). Rats were prepared for surgery by washing tail and shaving fur from the lower half of their body using an electric clipper, with a 0.25 mm blade (40SS CeramicEdge, Andis, Sturtevant, WI.). Immediately prior to surgery, the feet, legs, groin and abdomen were scrubbed with chlorhexidine soap and prepped with chlorhexidine clear solution. All surgery was performed under a mixture of isoflurane and oxygen anesthesia, and all efforts were made to minimize suffering.

### *In-Vivo* Pharmacokinetic Study

Rats (n = 4 per time-point) were used to evaluate the local (bone, muscle) and systemic (plasma) concentration of vancomycin after local intramedullary delivery to the rat tibia. While under general anesthesia, the right lower extremity was passed through a sterile half-drape and positioned away from the surgeon. A longitudinal incision was made with a sterile scalpel (#10), in the sagittal plane, extending 10 mm distally from the tibial tuberosity. A sterile sharp-ended Kirschner wire (0.054 in diameter) was used, at the proximal fourth junction, to make a hole through the cortical bone into the medial metaphysis and medullary canal of the tibia. The medullary cavity was then gently reamed with a 21G 1 1/2” needle, followed by reaming with an 18G 1” needle in order to reach the distal part of the tibial canal. The cavity of the medullary canal was excavated as much has possible by attaching a sterile syringe to the end of the 18G 1” needle and aspirating the contents 3–4 times before applying treatment. Gentle reaming provided clearance of intramedullary tissue/debris to allow for uniform filling of the medullary canal. Vancomycin hydrochloride solution (i.e., ~50 μL; 25 mg/Kg dose) was then injected into the canal using a sterile 21G needle and Hamilton syringe. The hole was then sealed with sterile bone wax and the incision was closed using stainless steel wound clips or simple stiches. Buprenorphine (i.e., 0.01–0.05 mg/Kg) was administered for analgesia upon emergence from anesthesia. Rats were given food and water *ad libitum* and monitored daily until defined time-points (i.e., 0 h, 4 h, 24 h, 48 h, 72 h, and 96 h) when they were sacrificed. Before sacrificing the rats, 800 μL of blood was drawn from their tail vein. At sacrifice, the tibia, and surrounding muscles were harvested and prepared for HPLC analysis. It should be noted that the medullary canal was reamed to remove any contents prior to analyzing the bone concentration. Therefore the bone concentration is a true representation of drug within the bone.

### HPLC Analysis

High performance liquid chromatography (HPLC) was used to detect vancomycin in bone, muscle, and plasma samples. For all tissue samples an Acclaim 120 C18 reverse phase column with 5μm particle diameter, 4.6 inner column diameter, and 250 mm in length (Thermo Fisher). Detection was done at 235 nm with an Agilent Technologies 1260 Infinity diode array detector at a temperature of 45°C. Bone extractions were done by adding 1% formic acid at 0.5g/mL to pulverized tissue. Samples were vortexed for 1 min and then centrifuged at 12,000 rpm for 10 min. The supernatant was injected after filtration with a 0.22 μm PES filter. Muscle extractions were done by adding mobile phase at 1.0 g/mL to pulverized tissue. Samples were vortexed for 1 min and then centrifuged at 15,000 rpm for 10 min. The supernatant was injected after filtration with a 0.22 μm PES filter. Plasma samples were diluted in mobile phase at a 1:1 ratio and injected as such. Calibration curves were done for each tissue type with blank matrix. Calibration curves were done by spiking known quantities of drug in blank matrix extracts followed by serial dilutions. The curve was linear in the range quantified from 1 to 16 μg/mL. Levels below 1 μg/mL were not detectable. All area under the curve (AUC) measurements were determined using Chemstation software Rev. B.04.03 (Agile Technologies, Santa Clara, CA). The mobile phase was a premixed solution of 90:10 50 mM KH_2_PO_4_ in 0.1% formic acid:acetonitrile. Adjustments were made with 0.1% formic acid in acetonitrile based on tissue type as needed.

### Preparation of Bacterial Inoculum

Bioluminescent strain *Staphylococcus aureus* Xen 36 (Caliper LifeSciences, Alameda, CA) was used to develop bone infection. The strain was stored in a glycerol (20% v/v) and Brain Heart Infusion (BHI) suspension at -80°C until required. Bacteria were subcultured onto an LB plate (Luria-Bertani, Miller broth mixed with 1.2% (w/v) Technical Agar (Difco, Sparks, MD.)), at 37°C overnight. Five colonies were then transferred to 3 mL LB broth and grown in shaker incubator (200 rpm), at 37°C for 2.5 h. Two milliliters of the bacterial suspension was transferred to 100 mL LB broth and grown for a further 4 h, until the cells reached exponential growth. Bacterial suspension was transferred into 50 mL centrifuge tubes and cells centrifuged at ~3,000 rpm for 30 min. Supernatants were discarded and pellets were re-suspended in 15 mL Phosphate Buffered Saline (PBS). Cells were then mixed by vortexing and the centrifugation process was repeated. This cell washing process was repeated three times. The final washed and pelleted cells were re-suspended in a total of 10 mL PBS, and mixed by vortexing. The cell suspension was then ten-fold serially diluted. The concentration of the cell suspension was then determined using the spread plate technique and spreading 100 μL of the various dilutions onto LB plates, in duplicate. Plates were incubated at 37°C overnight before counting colony forming units (CFU). The viable count was then used to dilute the washed cell suspension to the desired concentrations (i.e., average Log_10_ 7.82 CFU/mL or 6.62x10^7^ CFU/mL) using PBS. Two hundred microliter aliquots were dispensed into microfuge tubes and labeled bacterial inoculum. To inoculate the rat tibias the bacterial inoculum was mixed with equal volume of 1% (w/v) molten agarose (Pulsed field certified agarose, Biorad, Hercules, CA). Mixing the bacteria with agarose helped to control seepage of the inoculum and minimize contamination of the surrounding muscle.

### *In-Vivo* Infection Prevention Study

While under general anesthesia, a corticotomy was made in the tibia as described above, and 10 μL bacterial inoculum (i.e., average Log_10_ 7.82 CFU/mL in 0.5% w/v agarose) was added to medullary canal followed by vancomycin solution (i.e., ~ 50 μL; 25mg/Kg dose). The hole was then sealed with sterile bone wax (Sharpoint™, Surgical Specialties Corp.) and the wound was closed using 7 mm stainless steel wound clips (Reflex™ Skin Closure System, CellPoint Scientific Inc., Gaitherburg, MD) or simple stitches (S&T Microsurgical Suture w/suture thread, Fine Science Tools, Foster City, CA).

### *In-Vivo* Imaging

Images of the rats were taken using the IVIS Lumina II Imaging System (Caliper Life Science, Alameda, CA). Rats were sedated using isoflurane (1–5%) and oxygen (2 L/min) mixture during imaging. Rats were placed in the supine position inside the cabinet and placed with their right hind leg in the center of the field. The following parameters were used when taking the images: filed of view (FOV) = 12.5; binning = 8; f-stop = 1.0; and exposure time = 300 s. The luminescent color scale for all IVIS images was normalized to have a min and max radiance range of 3.48e4 and 3.48e5 (p/sec/cm^2^/sr), respectively.

X-ray images of the rat tibias were taken using the Kodak Carestream Imaging System (Carestream Health, Rochester, NY). Rats were sedated using isoflurane (1–5%) and oxygen (2 L/min) mixture during imaging. Rats were placed in the prone position inside the cabinet and placed with their right hind leg in the center of the field to visualize the whole tibia. The following parameters were used when taking the images: field of view (FOV) = 45.3 mm; focal point = 9.2 mm; f-stop = 9.18; X-ray filter = 0.8 mm; and exposure time = 180 s. These resulted in X-ray exposure = 35 kVp and X-ray current = 149 μA.

The 3D images of the rat tibias were taken using the Quantum FX Micro-CT System (PerkinElmer, Waltham, MA). Rats were sedated using isoflurane (1–5%) and oxygen (2 L/min) mixture during imaging. Rats were place in the supine position inside the cabinet. The scan parameter conditions used: kV = 90; CT μA = 160; field of view (FOV) = 60 mm; scan time = 120 s.

### Microbiological Analysis

After sacrificing the rats, the tibia and gastrocnemius muscle surrounding the surgical site were explanted, weighed separately and flash frozen with liquid nitrogen prior to pulverizing in a freezer mill (6770 Spex SamplePrep, Metuchen, NJ.). The powdered samples were then re-weighed, suspended (i.e., 1:10) in phosphate buffered saline (PBS), vortexed, and 10-fold serially diluted before plating (in duplicate) 100 μL of suspensions onto LB plates (Luria-Bertani, Miller broth mixed with 1.2% (w/v) Technical Agar (Difco, Sparks, MD.)). Plates were incubated at 37°C for 18–24 h and the bacterial load (colony forming units) per sample was calculated based on the number of colonies seen at specific dilutions. Confirmation of isolating *S*. *aureus* Xen 36 colonies from samples was determined using the IVIS (Lumina II, Caliper Life Science, Hopkinton, MA.). Non-bioluminescent colonies were confirmed as *S*. *aureus* using Gram staining, catalase test and Color Staph ID kit (Sure-Vue, Fisher Scientific, Houston TX.).

## Results

### *In-vivo* pharmacokinetics study

**Fig 1:**
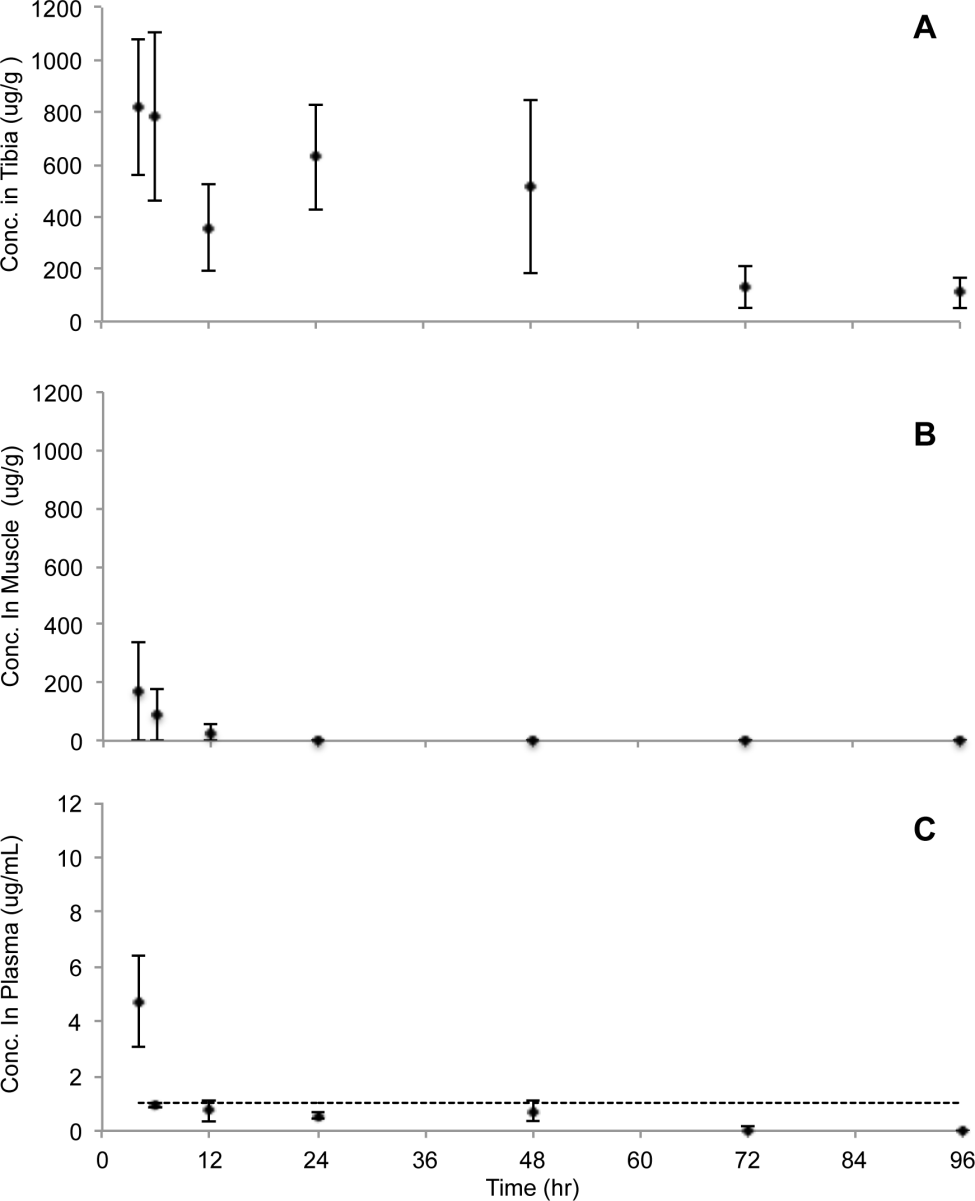
Pharmacokinetics of a single dose of vancomycin administered intraosseously into rat tibia. A 25 mg/Kg dose of vancomycin was delivered directly into the medullary canal of the tibia and a group of rats (n = 4) were sacrificed per time point. Study ended at 96-hours post initial administration. A supratherapeutic antimicrobial dose (i.e. >100 μg/mL) was maintained for up to 96 hours in the tibia (A), compared to < 6 hours in the surrounding muscle (B), while not exceeding systemic toxic levels (i.e. > 10 μg/mL) in the blood plasma (C). The mean vancomycin concentrations were obtained from 4 rats per time point, and error bars represent the standard error (SE).

Using HPLC analysis of pulverized tissue, high therapeutic concentrations (i.e. > 100 μg/g) of vancomycin were detected in the tibia over the 96-h study period (Fig 1), after a single dose of vancomycin hydrochloride solution (25 mg/Kg) to the medullary canal of the tibia. The mean vancomycin concentrations (± standard error) in the tibia were as follows: 818 μg/g (±261) (4h), 784 μg/g (±325) (6h), 359 μg/g (±166) (12h), 627 μg/g (±200) (24h), 515 μg/g (±332) (48h), 130 μg/g (±78) (72h), and 110 μg/g (±61) (96h). In comparison, vancomycin concentrations in the gastrocnemius muscle surrounding the surgical site only contained >100 μg/g for the first 4h post administration and dropped to near undetectable levels of vancomycin by 24h (Fig 1). The detectable vancomycin concentrations in the muscle were as follows: 168 μg/g (4h), 90 μg/g (6h), and 29 μg/g (12h). Little to no vancomycin was detected in the blood plasma of the rats for the entirety of the study (Fig 1). The only detectable vancomycin concentration in the plasma was 5 μg/mL (4h). (All raw data are presented in the Supporting Information section; S1 Table). The detection limit for vancomycin in the bone, muscle, and plasma samples using the HPLC procedure was 1 μg/mL. These results indicate that high concentrations of vancomycin hydrochloride solution, when delivered directly to a long bone, can remain locally within the bone. The number of rats (n = 4) used to determine the pharmacokinetics of locally delivered vancomycin over 96 hours, are based on previous pharmacokinetics studies [19, 20].

### *In-vivo* infection prevention study

**Fig 2:**
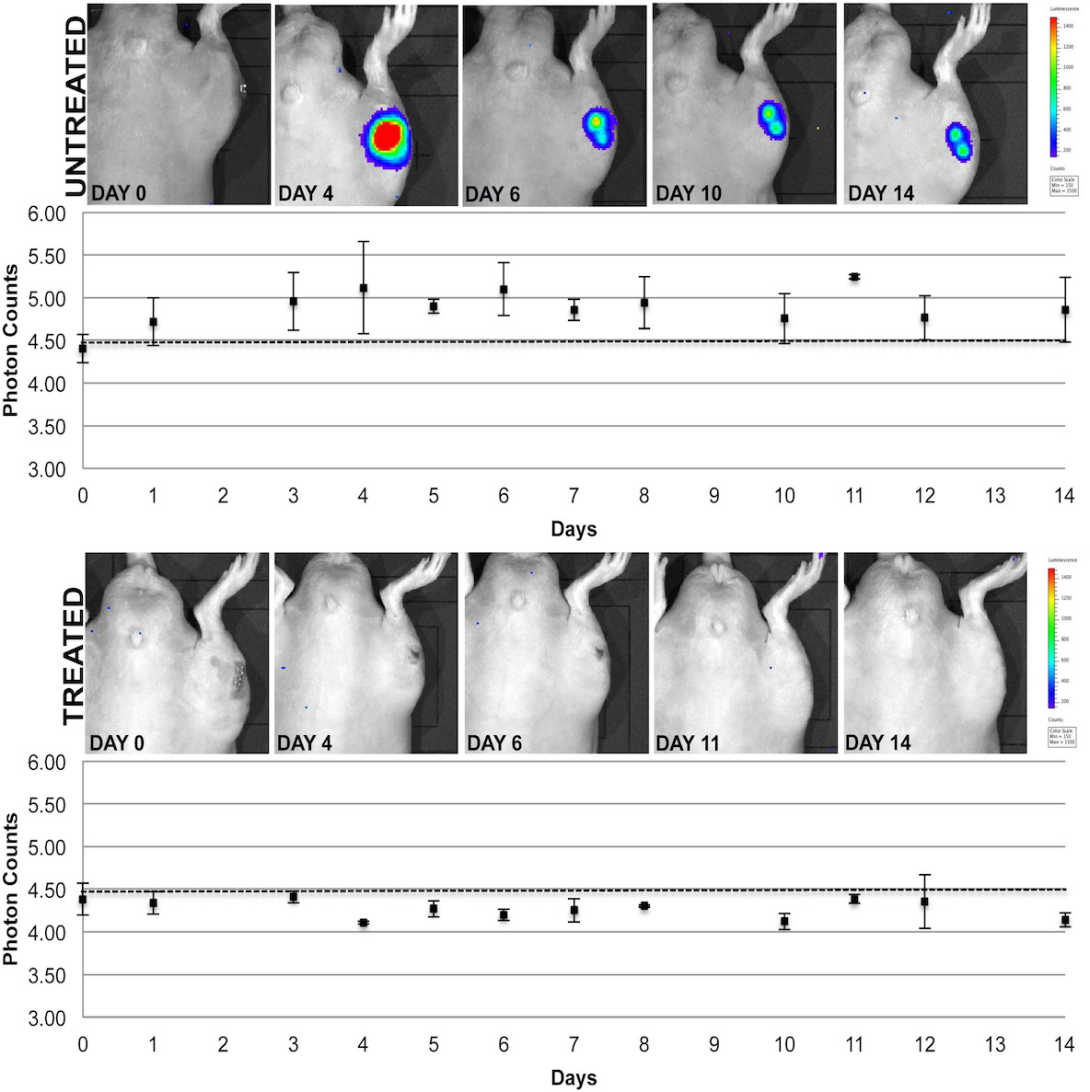
IVIS imaging of infected rats with and without the prevention treatment. The photon counts detected are displayed in the graphs above, along with images of representative rats showing bioluminescent intensity seen on specific days throughout the 14-day long study period.

**Fig 3:**
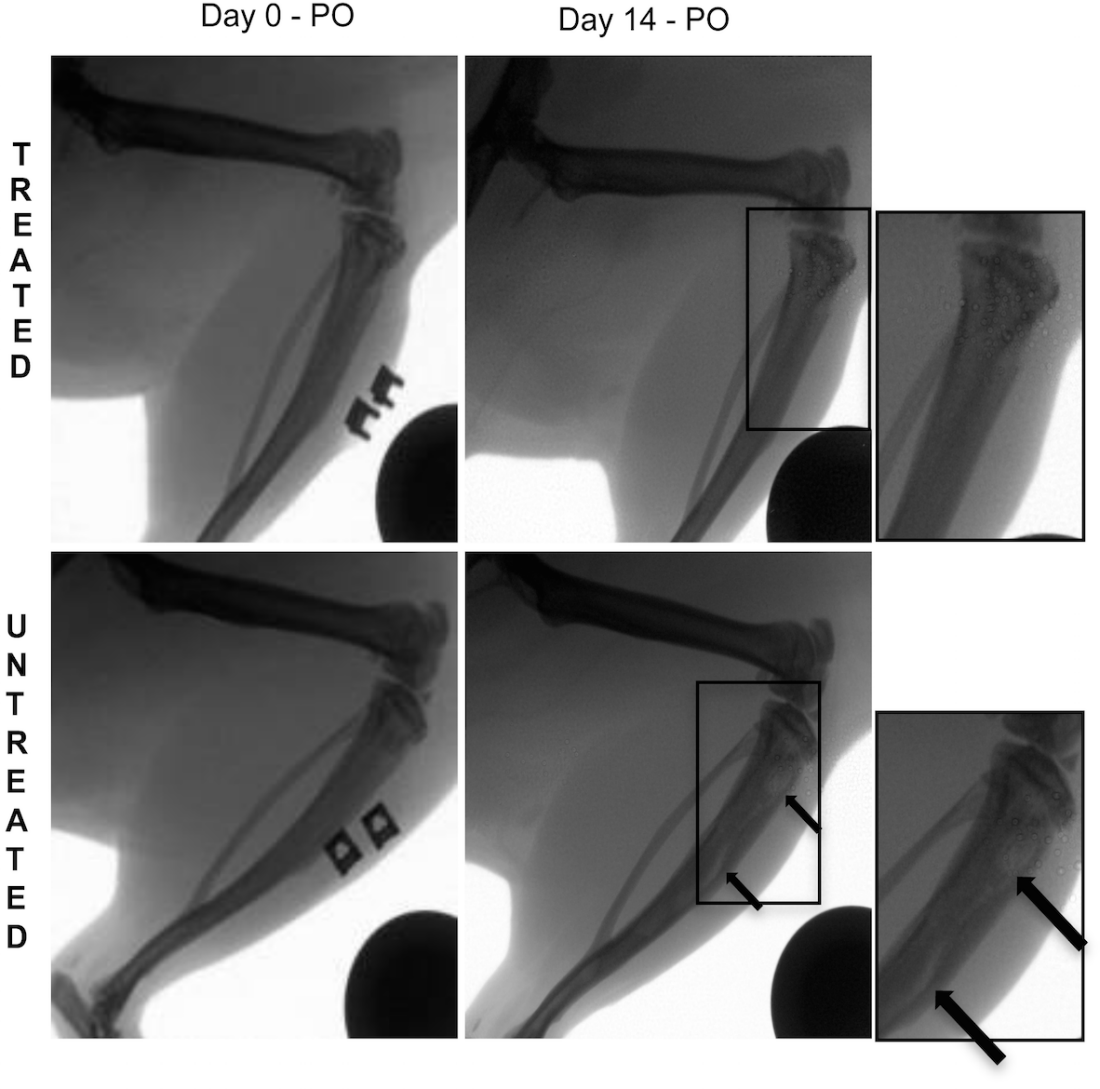
**X-ray images of rats in the treated group (top row) and untreated group (bottom row).** Radiolucency can be seen in the magnified X-ray image of the untreated rat tibia consistent with osteolysis, as highlighted by the black arrows.

**Fig 4:**
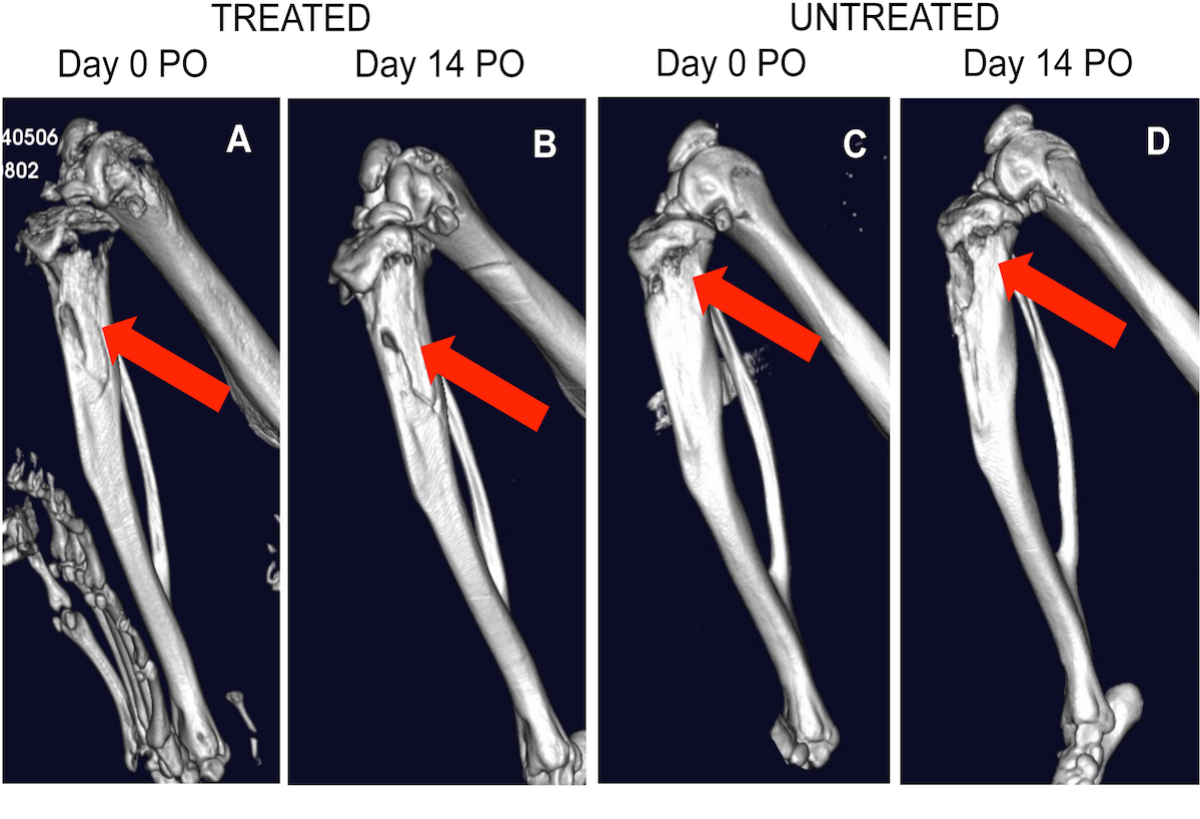
Micro-CT images of the right tibia of treated and untreated rats. Red arrows indicate the location of the hole made in order to introduce the bacterial inoculum into the medullary canal. Images show the hole for a treated rat at [A] day 0 and [B] day 14; and an untreated rat at [C] day 0 and [D] day 14 post surgery.

At the time of surgery, rats were inoculated with a bioluminescent *S*. *aureus* strain (Log_10_ 5.5 CFU/5 μL), with the treated group simultaneously administered a single dose of vancomycin (25 mg/Kg), compared to the untreated group, which were administered PBS as the placebo. All rats were then followed for 14 days during which time IVIS, X-ray, and micro-CT images were taken to compare differences between treated and untreated groups. At the end of the study period, all of the untreated rats (n = 6) developed radiographic evidence of tibial osteomyelitis while zero of the vancomycin treated rats (n = 9) displayed radiographic signs of osteomyelitis. Fig 2 shows IVIS images of metabolically active bioluminescent *S*. *aureus* present in the right hind leg of an untreated rat at days 1, 5 and 14, compared to its absence in a treated rat (All raw data are presented in the Supporting Information section; S2 Table). Fig 3 shows representative X-rays images of an untreated rat with radiolucency in its right tibia, as highlighted by the black arrows, compared to a treated rat with little to no osteolysis occurring in its tibia. Fig 4 shows micro-CT images (A and B) of the tibia of a treated rat, which shows the initial surgical hole reduced in size by day 14, compared to Fig 4 micro-CT mages (C and D), which show the surgical hole in the tibia of the untreated rat increased in size by day 14.

## Discussion

Vancomycin has traditionally been administrated via intravenous infusion for the treatment of orthopaedic infections. Its elimination half-life in a patient with normal renal function is approximately 6 hours with a narrow therapeutic index [11, 21]. Certain classes of antimicrobials exhibit concentration dependent killing and others exhibit time dependent killing. Vancomycin tends to display a mixture of the two types but is predominantly time-dependent; therefore, it works best when the concentration of the drug is greater than the MIC >50% of the dosing interval [22, 23]. Vancomycin is a glycopeptide and is considered bacteriocidal against MRSA. The MIC of vancomycin for MRSA typically falls between 1 and 2 mg/L. High (sustained) trough concentrations of the drug can result in nephrotoxicity and ototoxicity; therefore, in individuals with normal renal function, troughs between 10–20 mg/L should be targeted. The ideal dosing strategy involves achieving the highest safe concentration for the longest period of time [24, 25]. The Infectious Diseases Society of America, The American Society of Health-System Pharmacists, and the Society of Infectious Diseases Pharmacists recommend that the initial vancomycin dosage used should be based on the patient’s body weight. To rapidly attain this target concentration, a loading dose of 25–30 mg/Kg is required [26]. Our method of locally delivering vancomycin to the medullary canal of long bones has shown that it is possible to obtain therapeutic or supra-therapeutic bone concentrations of vancomycin (i.e., >100 ug/mL) yet maintain a very low, almost undetectable, concentration of vancomycin in the surrounding tissues and systemic circulation.

Vancomycin has been used to treat osteomyelitis and soft tissue infections for several decades. Although not specifically approved by the Food and Drug Administration (FDA) to treat bone and joint infection, it is approved to treat serious infections caused by MRSA [21]. The treatment duration tends to depend on the type of infection being treated: soft tissue infections commonly require 2 weeks; intra articular infections require 2 to 4 weeks; and osteomyelitis requires 4 to 6 weeks. For institutions where the prevalence of MRSA is high, vancomycin is recommended for perioperative prophylaxis in patients undergoing orthopaedic surgical procedures. It is also recommended for patients who are presumed or colonized with MRSA [2, 7]. The use of prophylactic antimicrobial agents during orthopaedic surgical procedures, such as hip arthroplasty, is an effective method for reducing the rate of postoperative wound infection [27].

**Fig 5:**
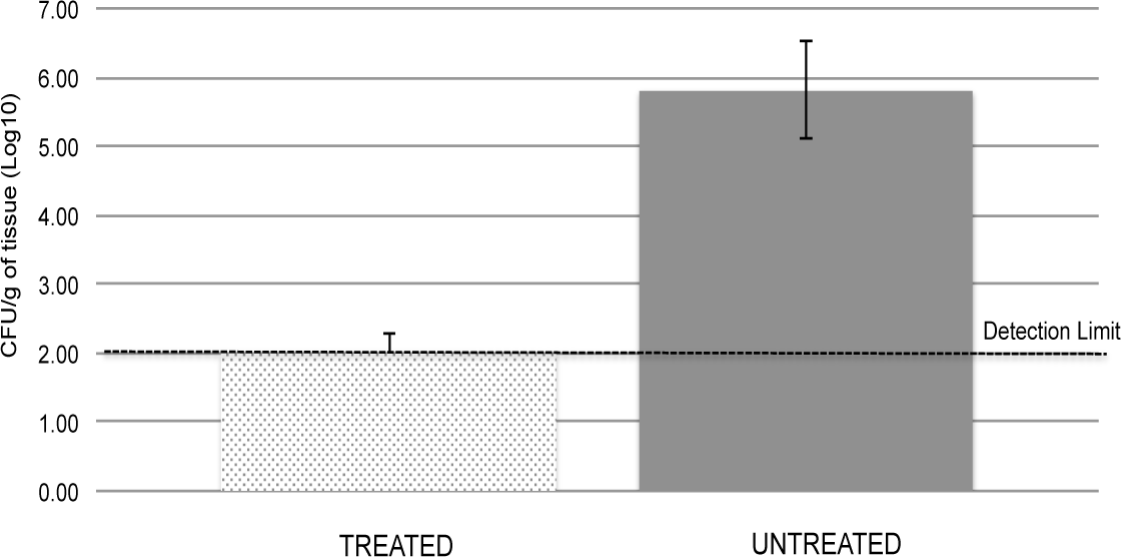
Bacterial load of tibia explanted from the infected rats with and without prevention treatment. Microbiological analysis of explanted tibias revealed the presence of viable bacteria (average Log_10_5.8 CFU/g) in the untreated rats, compared to undetectable levels of bacteria (< 200 CFU/g) in the treated rats. No bacteria were detected in the muscle and plasma samples of the treated or untreated rats at the end of the study.

To determine if the prophylactic administration of a single dose of vancomycin (25 mg/Kg) could prevent infection and the development of osteomyelitis, rats that underwent a corticotomy of the medial metaphysis into the medullary canal of the tibia, were inoculated with a bioluminescent *S*. *aureus* strain (Log_10_ 5.5 CFU/5 μL) and simultaneously administered intramedullary vancomycin (~ 50 μL; 25 mg/Kg dose), and were then followed for 14 days (S3 Table). At the end of our study, rats that had been given the prophylactic treatment showed no signs of infection around the surgical site, and did not contain detectable levels (i.e. <200 CFU/g) of viable bacteria in their tibia (Fig 5; S4 Table). This pre-clinical animal study supports the use of locally delivered vancomycin as an effective prophylactic treatment to prevent the development of osteomyelitis by MRSA. This potential treatment fits well with the use of antibiotics for prophylaxis to reduce surgical infection rates [7], or conversion of a contaminated open fractures to osteomyelitis, which are found to have an infection rate of ~2.5% [28].

A single high local dose of antibiotic at the surgical site may reduce the need for prolonged prophylaxis protocols, which is the current standard of practice for patients colonized with MRSA [8]. Patients admitted to the hospital who are colonized by MRSA (~ 5%) [29] have an even higher risk of developing MRSA infection after surgery [8]. Our method of directly prophylaxing the bone may be beneficial in orthopaedic surgeries for the prevention of methicillin-resistant *Staphylococcus aureus* infections [30]. This animal model is not designed to show how effective our local treatment would be after osteomyelitis has developed. However, we are conducting future studies to test the effectiveness of our local treatment in treating established osteomyelitis by administering the antibiotic at 14-days post bacterial inoculum, once bone infection has been confirmed using radiographic and bioluminescent imaging. For appropriate osteomyelitis rat models, readers are directed to review the following papers: [31–33].

The ability to use state-of-the-art non-invasive imaging systems such as the bioluminescent imaging technology like the IVIS to detects bioluminescent bacteria *in vivo* in real-time [34, 35], and microfocus computed tomography (micro-CT) like the Quantum FX system, used to assess a three-dimensional architecture of adaptive bone (re)modeling during infection [36], has allowed us to limit the number of animals needed to conduct our pre-clinical studies, while providing qualitative and quantitative analysis to compare our treated and untreated groups. Micro CT evaluation showed that the treated rats had improved bone remodeling and were absent of any signs of osteomyelitis compared to the untreated controls.

Although the administration of locally delivered vancomycin, specifically in its powdered form, is becoming increasingly popular with surgeons to reduce the development of surgical site infections [37], further pre-clinical work is needed to determine the effects of high concentrations of vancomycin on the surrounding tissue. To date, a handful of studies have investigated the detrimental effects of high vancomycin concentrations on various types of cell lines. Edin et al. showed that vancomycin concentrations of 10 mg/mL caused cell death in the osteosarcoma cell line MG-63 *in vitro* [38]; Antoci et al. found doses of greater than 2 mg/mL severely decreased cellular proliferation of osteoblast (MC3T3-E1) and chondrocyte (N1511) cells involved in bone healing [39]; and Yoeruek et al. demonstrated that vancomycin concentrations higher than 5 mg/mL led to significant reductions in the viability of human corneal endothelial cells, to near total destruction over 10 mg/mL [40]. Our maximum concentration is an order of magnitude lower than these reported toxic levels.

A limitation to our study is using small rodents to determine the pharmacokinetics of locally delivered vancomycin. The rate of drug elimination is faster in rodents than in humans [41], and our dosing results would need to be reevaluated in humans. Another limitation to our study is the use of a single isolate of *S*. *aureus*, which may produce different results to MRSA isolates found in different patients [42, 43].

## Conclusion

This study has provided us with the following insights: 1) The infused drug stays in the bone for a long period of time (i.e. at least 96 hours); 2) the blood and muscle surrounding the tibia were exposed to very low concentrations of vancomycin over the 96 hour period; and 3) a potential dosing interval for repeat-dosing experiments could be administered as frequently as every 96 hours. We were initially concerned that due to the highly vascular environment and communication between the intramedullary space and the systemic circulation, the drug would leave the bone very quickly and raise the systemic concentration to toxic levels, but this was not the case.

Our studies have shown that intramedullary delivery of vancomycin results in high local drug concentrations with minimal systemic vancomycin concentrations. We have also shown that concurrent delivery of vancomycin and *S*. *aureus* to the tibial intramedullary canal can prevent the development of osteomyelitis compared to untreated controls. Future studies will be conducted to determine if this technique could be used as a treatment strategy for bone infections.

## Supporting Information

S1 Table HPLC analysis of detectable vancomycin in tibia, muscle, and plasma after intramedullary delivery in rats. (XLSX)

S2 Table Bioluminescent activity for rats that underwent prevention study. (XLSX)

S3 Table Bacterial inoculum used for prevention study. (XLSX)

S4 Table Microbiological analysis of explanted tissue samples from the infected rats with and without prevention treatment. (XLSX)
